# Reduction of Strangulated Herniæ by Coughing

**Published:** 1890-03-08

**Authors:** 


### SURGERY.

### REDUCTION OF STRANGULATED HERNIÆ BY COUGHING.

Dr. Vandenabule, of Strassburg, was endeavouring to reduce by taxis a strangulated inguinal hernia induced by severe and constant coughing in a patient suffering from pulmonary emphysema. The hernia was the size of an orange, and he had been exerting taxis for twenty minutes without result when the man coughed violently. Dr. Vandenabule did not release his grasp but heard a gurgling and found that the sac was much reduced in bulk. This fit of coughing had been involuntary, but after a few minutes' rest he again seized the sac and directed the man to cough again. While doing so it returned entirely. He believes that in the act of coughing both the inguinal and crural rings are dilated, and that hernia is thus induced, but that by exerting support and pressure on the contents of the sac, one may avail oneself of the same momentary dilatation of the ring for their reduction. Having employed this method successfully in ten cases of inguinal hernia in men, and seven of femoral in women, he thinks that is deserving of more general attention.

